# Diabetic kidney disease—Recent updates

**DOI:** 10.1111/1753-0407.13612

**Published:** 2024-08-18

**Authors:** Zachary Bloomgarden

**Affiliations:** ^1^ Department of Medicine, Division of Endocrinology Diabetes and Bone Disease, Icahn School of Medicine New York USA

The ramifications of the effects of diabetes on the kidney and the relationships of renal disease to the complications of diabetes are manifold, and several recent studies have addressed important aspects of the implications and the management of diabetic kidney disease (DKD).

An estimate of the prevalence of DKD among persons with type 1 diabetes (T1D) was made based on the National Health and Nutrition Examination Survey (NHANES) database of 19 225 adults in the United States from 2015 to 2018; 47 had T1D, among whom 20 had estimated glomerular filtration rate (eGFR) ≤60 mL/min/1.73 m^2^ or urine albumin/creatinine ratio (UACR) ≥30 mg/g, allowing estimates of 1 202 739 adults in the United States with T1D and a weighted estimate that 21.5% of people with T1D in the United States have DKD.^1^ A report from the Centers for Disease Control Wide‐Ranging Online Data for Epidemiologic Research database mortality statistics from 1999 to 2020 reflected the dramatic increase in mortality associated with DKD; more than 500 000 deaths were reported among adults with DKD during this period, with an age‐adjusted annual mortality rate per 100 000 persons of approximately 2.0 in 1999–2005, increasing to approximately 4.0 in 2007–2010, but then to 22.0 in 2012–2019 and to 25.0 in 2020.^2^ In an analysis suggesting interrelationships between DKD and cognitive function (CF), among 2977 people with type 2 diabetes (T2D) in the Action to Control Cardiovascular Risk in Diabetes (ACCORD) memory in diabetes trial, there was a greater decline over 40 months in CF with standard than with intensive glycemic treatment among those with urine albumin <0.4 mg/dL, whereas those with higher levels of albuminuria had no evidence of benefit with intensive treatment, and for those with eGFR < 60, CF decline was greater with intensive than with standard glycemic treatment. Similarly, CF decline was greater with standard than intensive glycemic treatment in the subset age <60 years, suggesting that T2D with better renal function and lower age might particularly benefit from more intensive glycemic treatment.^3^ There may be a different relationship between age and renal outcome with intensive lifestyle intervention (ILI); in a 12‐year follow‐up of the Look AHEAD (Action for Health in Diabetes) trial, prespecified analysis of the relationship between the ILI and age showed that among 5112 participants with baseline eGFR ≥ 45, those aged >60 years at baseline randomized to ILI had a 25% lower likelihood of eGFR decreasing to <45 mL/min/1.73 m^2^, whereas this was not seen in the younger participants.^4^

The optimal blood pressure treatment target is still not certain. The 11 255‐person Effects of Intensive Systolic Blood Pressure Lowering Treatment in Reducing Risk of Vascular Events (ESPRIT) trial included 4359 persons with diabetes with systolic blood pressure (BP) decreasing from baseline of 147 to on‐trial mean systolic of 119 (intensive) versus 135 (standard) followed for 3.4 years; the combined end point of myocardial infarction (MI), stroke, heart failure (HF), death, and revascularization occurred significantly less often with the intensive than the standard BP goal, in 9.7% versus 11.1% of the enrolled persons; there was a particularly great and statistically significant 39% reduction in the CV mortality with intensive BP treatment, and similar between‐group differences of MI, HF, and stroke, without heterogeneity of effects by the presence or duration of diabetes.^5^ Renal function declined, however, in 3.0% of intensive versus 1.8% of the control participants,^6^ a potential concern as similar reduction was reported with intensive BP‐lowering in the ACCORD Blood Pressure trial and in the Systolic Blood Pressure Intervention Trial (SPRINT).^7^

There is extensive evidence of the renal protection benefits of treatment with angiotensin receptor blockers (ARB) and angiotensin converting enzyme inhibitors (ACEi) in people with DKD, but these agents are often discontinued due to worsening renal function. A recent individual patient meta‐analysis of 18 eligible studies addressed the important issue of the use of these agents with macroalbuminuric chronic kidney disease stages 4 and 5: Among 1739 participants with eGFR <30 (median: 23), with median UACR: 1215, ACEi or ARB led to 34% lower risk of kidney failure or renal transplant end‐stage kidney disease (ESKD), with similar benefit in those with versus without diabetes.^8^ An intriguing recent study suggests the nephroprotective effect of metformin in DKD; at follow‐up of 137 514 newly diagnosed T2D patients from 2007 to 2016, using a propensity score matching approach, doubling of serum creatinine sustained for at least 3 months was 29% lower, the incidence of GFR ≤ 15 mL/min/1.73 m^2^ was 39% lower, and the incidence of ESKD was 45% lower in those receiving metformin, controlling for factors including baseline creatinine, HbA1c, and other medications.^9^

Use of sodium‐glucose cotransporter‐2 inhibitors (SGLT2i) has protective effect in patients with DKD.^10^ The SGLT1/2 inhibitor sotagliflozin has such effect in patients with T1D, lowering UACR along with initial reduction eGFR,^11^ and a study of 10 584 patients with T2D and DKD now shows sotagliflozin to be associated with a 33%–40% lower likelihood of ≥50% decline in eGFR, of kidney failure, of fall in eGFR to <15, and of need for dialysis or transplantation.^12^ SGLT2i appear to reduce the likelihood of hyperkalemia. A propensity score‐matched comparison of adults with T2D newly starting SGLT2i versus dipeptidyl peptidase 4 inhibitors (DPP4i) versus glucagon‐like peptide‐1 receptor agonists (GLP‐1RA) used a dataset of >700 000 persons with each agent, finding hyperkalemia incidence rates of 25.3 versus 18.5 comparing SGLT2i versus DPP4i, of 28.5 versus 22.1 comparing GLP‐1 RA versus DPP4i, and of 22.1 versus 19.8 events/1000 person‐years comparing GLP‐1RA versus SGLT2i, respectively.^13^ In a network meta‐analysis of 27 studies involving 43 589 participants with DKD, combined use of SGLT2i and ACEi/ARB was associated with 61% lower risk of hyperkalemia than ACEi/ARB alone.^14^ There is increasing interest in the use of mineralocorticoid antagonists (MRA), with a recent comparative analysis suggesting similar benefit of the nonsteroidal MRA finerenone in efficacy to that of the SGLT2i canagliflozin in its effect on development of end‐stage kidney disease, as well as showing similar effects on total and CV (cardiovascular) mortality, although with the MRA associated with greater rather than lower likelihood of hyperkalemia.^15^

The GLP‐1RA semaglutide was studied in the FLOW (evaluate renal Function with semagLutide Once Weekly) trial of 3533 persons with T2D with eGFR 50–75 and UACR > 300, or with eGFR 25–50 and UACR > 100. Those randomized to semaglutide 1 g daily versus placebo had 5.8 versus 7.5 renal events (≥50% decrease in eGFR, kidney failure, or kidney failure mortality) per 100 patient‐years, giving a number‐needed‐to‐treat (NNT) of 20, as well has having 18% and 20% decreases in CV events and in mortality, for NNTs of 45 and 39.^16^ A meta‐analysis of 12 trials of SGLT2i studied the effect of (nonrandomized) use of GLP‐1RA in addition to SGLT2i, showing a > 50% lower slope of eGFR reduction in those receiving both agents.^17^ Conversely, in the FLOW trial, 550 participants took SGLT2i at baseline; such treatment was associated with a numerically smaller reduction in eGFR at 104 weeks, both based on serum creatinine and on cystatin C, although the interaction was not statistically significant.^18^ A population dataset analysis of 6696 patients who started GLP‐1 receptor agonists and added on SGLT2i and 8942 patients who started SGLT‐2 inhibitors and added on GLP‐1 receptor agonists, propensity score‐matched in a 1:1 ratio to patients prescribed the same background drug, showed 57% lower likelihood of serious renal events with SGLT2i added to GLP‐1RA and 33% lower likelihood of serious renal events with GLP‐1RA added to SGLT2i.^19^

In summary, more than one in five adults with T1D in the US has DKD, and US mortality rates associated with DKD in T2D increased more than 10‐fold from 1999 to 2020. Intensive glycemic control might have greater cognitive function benefit in younger persons with T2D having better renal function, but intensive lifestyle intervention might have greater renal function benefit in older persons with T2D.

Although improving cardiovascular outcomes, intensive blood pressure reduction may be associated with worse renal function. ACEI/ARB are associated with renal benefit even withe GFR<30. The use of MRA may offer additional renal benefit, albeit with risk of hyperkalemia. SGLTi and GLP‐1RA are associated with renal benefit, and, particularly for SGLT2i, with lower likelihood of hyperkalemia. Finally, metformin may be associated with renal benefit.
